# Ellagic Acid Prevents Binge Alcohol-Induced Leaky Gut and Liver Injury through Inhibiting Gut Dysbiosis and Oxidative Stress

**DOI:** 10.3390/antiox10091386

**Published:** 2021-08-30

**Authors:** Dong-ha Kim, Yejin Sim, Jin-hyeon Hwang, In-Sook Kwun, Jae-Hwan Lim, Jihoon Kim, Jee-In Kim, Moon-Chang Baek, Mohammed Akbar, Wonhyo Seo, Do-Kyun Kim, Byoung-Joon Song, Young-Eun Cho

**Affiliations:** 1 Department of Food and Nutrition, Andong National University, Andong 36729, Korea; a960112@naver.com (D.-h.K.); yejin5879@naver.com (Y.S.); sayseven01@naver.com (J.-h.H.); iskwun@andong.ac.kr (I.-S.K.); 2 Department of Biological Science, Andong National University, Andong 36729, Korea; jhlim@andong.ac.kr; 3 Parker H. Petit Institute for Bioengineering and Bioscience, Georgia Institute of Technology, Atlanta, GA 30332, USA; jkim3441@gatech.edu; 4 Department of Biochemistry and Cell Biology, School of Medicine, Kyungpook National University, Daegu 41944, Korea; genekim10@gmail.com; 5 Department of Molecular Medicine, School of Medicine, Cell & Matrix Research Institute, Kyungpook National University, Daegu 41944, Korea; mcbaek@knu.ac.kr; 6 Division of Neuroscience and Behavior, National Institute on Alcohol Abuse and Alcoholism, Bethesda, MD 20892, USA; mohammed.akbar@nih.gov; 7 College of Pharmacy, Graduate School of Pharmaceutical Sciences, Ewha Womans University, Seoul 03760, Korea; wonhyoseo@ewha.ac.kr; 8 Korea Zoonosis Research Institute, Jeonbuk National University, Iksan 54531, Korea; dkkim714@jbnu.ac.kr; 9 Section of Molecular Pharmacology and Toxicology, Laboratory of Membrane Biochemistry and Biophysics, National Institute on Alcohol Abuse and Alcoholism, National Institutes of Health Bethesda, Bethesda, MD 20892, USA

**Keywords:** binge alcohol, ellagic acid, gut microbiota, intestinal barrier dysfunction, endotoxemia, inflammatory fatty liver injury

## Abstract

Alcoholic liver disease (ALD) is a major liver disease worldwide and can range from simple steatosis or inflammation to fibrosis/cirrhosis, possibly through leaky gut and systemic endotoxemia. Many patients with alcoholic steatohepatitis (ASH) die within 60 days after clinical diagnosis due to the lack of an approved drug, and thus, synthetic and/or dietary agents to prevent ASH and premature deaths are urgently needed. We recently reported that a pharmacologically high dose of pomegranate extract prevented binge alcohol-induced gut leakiness and hepatic inflammation by suppressing oxidative and nitrative stress. Herein, we investigate whether a dietary antioxidant ellagic acid (EA) contained in many fruits, including pomegranate and vegetables, can protect against binge alcohol-induced leaky gut, endotoxemia, and liver inflammation. Pretreatment with a physiologically-relevant dose of EA for 14 days significantly reduced the binge alcohol-induced gut barrier dysfunction, endotoxemia, and inflammatory liver injury in mice by inhibiting gut dysbiosis and the elevated oxidative stress and apoptosis marker proteins. Pretreatment with EA significantly prevented the decreased amounts of gut tight junction/adherent junction proteins and the elevated gut leakiness in alcohol-exposed mice. Taken together, our results suggest that EA could be used as a dietary supplement for alcoholic hepatitis patients.

## 1. Introduction

Alcohol-associated medical and socioeconomic burdens still pose serious problems in many countries, although these stigmas can be prevented by simple behavioral modification and abstinence. Indeed, the Centers for Disease Control and Prevention (CDC) reported that the annual economic loss regarding alcohol-related problems in the USA alone accounted for approximately $249 billion dollars in 2010 and that ~77% of alcohol-induced tissue damage is associated with binge drinking [1]. The clinical spectrum of alcoholic liver disease (ALD) includes alcoholic fatty liver (steatosis, AFLD), steatohepatitis (inflammation, ASH), fibrosis/cirrhosis, and increased risk of hepatocellular carcinoma [2,3]. In addition, recent reports demonstrate the critical roles of gut dysbiosis and subsequent intestinal barrier dysfunction (i.e., leaky gut) in inflammatory tissue injury caused by alcohol misuse [4,5] and nonalcoholic substances or pathological conditions [6,7]. In fact, excessive alcohol intake can alter the composition and abundance of gut microflora (gut dysbiosis) and intestinal permeability, contributing to elevated endotoxemia and endotoxin lipopolysaccharide (LPS), which then accelerates inflammatory damage to many organs, including steatohepatitis and neurodegeneration in rodents and humans, through the gut–liver–brain axis [5,6,7,8,9]. Alcohol-induced gut leakiness is critically important in the progression of ALD to more severe disease stages, including fibrosis/cirrhosis since the elevated levels of endotoxin are positively correlated with the development of liver cirrhosis [10]. Unfortunately, many patients with alcoholic hepatitis are known to die within 30~60 days after their diagnoses and high serum LPS levels are critical determinants of alcohol-mediated inflammatory multi-organ failure and deaths [11]. This clinically challenging condition suggests an urgent need for the development of safe and effective therapeutics and/or preventive agents against ASH. One potential approach to help in managing the severity of ASH can be achieved by preventing gut barrier dysfunction and elevated endotoxemia with dietary supplements and/or the potential repurposing of the existing drugs that were already approved by the Food and Drug Administration (FDA).

Ellagic acid (EA), a natural polyphenolic compound, is found in many gallnuts and fruits, such as raspberries, strawberries, cranberries, grapes, black currants, pomegranate, and mango [12]. Previous studies have reported that EA possesses several biological properties, such as antidiabetic, anti-inflammatory, and antioxidant activities. EA also inhibits the activation of hepatic stellate cells and mast cells, the proliferation of transformed cells, as well as viral replication by increasing antioxidant response, induction of apoptosis, downregulation of the genes involved in cell cycle and angiogenesis, and the stimulation of the cellular immune response [12]. Indeed, our previous report revealed that a pharmacologically high dose of dried pomegranate extract containing 40% EA prevents alcohol-induced gut leakiness and alcoholic hepatitis in rats, as well as the apoptosis and epithelial permeability of cultured T84 colon cells [13]. Our recent results also showed that alcohol-mediated gut leakiness and steatohepatitis depended on oxidative stress, which is, at least partially, produced by the ethanol-inducible cytochrome P450-2E1 (CYP2E1) in the intestines and liver, since *Cyp2e1*-null mice were protected from gut leakiness and inflammatory liver injury despite exposure to very high doses of alcohol [14]. However, the effects of a physiologic-relevant dose of EA (approximately 60 mg/kg/day in mice) on alcohol-induced gut dysbiosis, leaky gut, endotoxemia, and liver injury in rodents have not been known or systematically studied. In this study, we investigated the effects of different EA doses (e.g., 10, 30, 60, or 90 mg/kg/day) on binge alcohol-mediated gut leakiness and liver injury. We specifically tested a hypothesis that a physiologically relevant dose of EA can avert binge alcohol-induced leaky gut by stabilizing the composition and abundance of gut microbiota and/or suppressing CYP2E1-mediated oxidative stress in the gut and liver, contributing to the prevention of intestinal barrier dysfunction, endotoxemia, and inflammatory fatty liver disease.

## 2. Materials and Methods

### 2.1. Materials

EA used in this study was purchased from Sigma Chemical (St. Louis, MO, USA). Other chemicals and materials not described here were the highest grades available and/or the same, as recently described [13,14,15,16,17].

### 2.2. Animal Treatments

All animal experimental procedures were carried out by following the guidelines by National Institutes of Health (NIH) for small animal experiments and approved by the Andong National University Animal Care and Use Committee. All mice were maintained under controlled lighting (12 h light/dark cycle) with food and water provided *ad libitum*. Age-matched 6-week-old female C57BL/6J mice (purchased from ORIENT BIO Inc. Seongnam-si, Korea) were subjected to oral administration of a daily dose of 60 mg/kg EA, freshly prepared by suspension in water just before treatment, based on the calculation for physiologically and clinically relevant doses [18] or silymarin (SM) 200 mg/kg/day (used as a positive control) [19]. Control mice were treated similarly by daily oral administrations with a vehicle (water). After EA pretreatment for 14 consecutive days, different groups of mice (*n* ≥ 4/group) were exposed to 3 oral doses of binge alcohol (5 g/kg/dose) or dextrose (as a control) at 12 h intervals and euthanized 1 h after the last ethanol dose for collecting various tissues and plasma, the same as recently described [13,14].

### 2.3. Histological Analysis and Plasma ALT Measurement

In this study, part of the largest liver lobe and small intestine (ileum) from each mouse exposed to EA, SM, or water pretreatment with or without binge ethanol exposures were fixed in neutral formalin. Paraffin-embedded blocks of formalin-fixed individual liver and ileum were cut at 4 microns and then stained with hematoxylin & eosin (H&E) by the Kyungpook National University (KNU) core lab. To further support fat accumulation, frozen liver samples embedded in optimal cutting temperature compound were cut (10 µm) and stained with Oil Red O by the KNU core lab. The plasma ALT level in each mouse was determined by using the standard end-point colorimetric assay kit (BioVision, Milpitas, CA, USA), as described [16].

### 2.4. Endotoxin Assay

Plasma endotoxin (LPS) levels were determined using the commercially available endpoint LAL Chromogenic Endotoxin Quantitation Kit with a detection range of 0.015–1.2 EU/mL (Thermo Fisher Scientific, Waltham, MA, USA), as previously described [13,14].

### 2.5. Determinations of Hepatic Triglyceride, Plasma Reactive Oxygen Species, and Blood Alcohol Concentration

The amounts of hepatic triglyceride (TG) were assessed by using a commercially available kit (Asan Co., Ltd., Gimpo, Korea). The quantities of plasma reactive oxygen species (ROS) were measured with 2′,7′-dichlorofluorescein diacetate (DCFH-DA, Thermo Fisher Scientific). Following incubation with DCFH-DA at 37 °C for 20 min, the DCFH-DA fluorescence was then determined by the method, as recently described [17]. The concentrations of blood alcohol concentration (BAC) were determined with a commercially available kit (BioVision) by following the manufacturer’s instructions [13,15].

### 2.6. Enzyme-Linked Immunosorbent Assay (ELISA)

The amounts of TNF-α, IL-1β, and CYP2E1 in the lysates of small intestines or livers were determined by using the respective ELISA kit (Abcam, Cambridge, UK) and CYP2E1 (Cloud-Clone Corp, Houston, TX, USA), respectively, by following the manufacturers’ protocols. The protein concentration was measured with the BCA reagent (BioRad, Hercules, CA, USA) to use equal amounts of protein for the ELISA. Duplicate samples from different lysates (*n* = 4/group) were used for ELISA, which was repeated twice.

### 2.7. Immunoblot Analysis

Parts of liver tissue or small intestine from each mouse were homogenized with 1× RIPA buffer. The same amounts (50 µg protein equally pooled from 4~5 different mouse tissues within the same group) were separated by SDS/PAGE and transferred to nitrocellulose membranes. These membranes were incubated with the respective rabbit polyclonal antibody against CYP2E1 (1:5000 dilution; Abcam), p-JNK (1:1000 dilution; Cell Signaling), 3-NT (1:5000 dilution; Abcam), iNOS (1:5000 dilution; Abcam), cleaved caspase-3 (1:1000 dilution; Cell Signaling), ZO-1 (1:5000 dilution; Abcam), or β-catenin (1:1000 dilution; Santa Cruz Biotechnology, Dallas, TX, USA). The specific mouse monoclonal antibody against JNK (1:1000 dilution; Cell Signaling), Bax (1:1000 dilution; Santa Cruz Biotechnology), β-actin (1:10,000 dilution; Santa Cruz Biotechnology), γ-catenin (1:1000 dilution; Santa Cruz Biotechnology), E-catenin (1:1000 dilution; Santa Cruz Biotechnology), occludin (1:1000 dilution; Santa Cruz Biotechnology), claudin-1 (1:1000 dilution; Abcam), or glyceraldehyde 3-phosphate dehydrogenase (GAPDH) (1:10,000 dilution; Santa Cruz Biotechnology) was also used to detect the respective target protein, as indicated. After washing the nitrocellulose membranes with PBS three times at 10 min intervals, horseradish peroxidase (HRP)-conjugated anti-rabbit or anti-mouse IgG (Santa Cruz Biotechnology) was used as the secondary antibody at 1:5000 dilution. Relative protein images were determined by using HRP-conjugated secondary antibodies and ECL substrates (Thermo Fishers). The intensities of the immunoreactive target protein bands relative to GAPDH used as a loading control were quantified by densitometry using the ImageJ software available from NIH.

### 2.8. Apoptosis Assay

Small intestine and liver specimens were fixed overnight in 10% buffered neutral formalin and embedded in paraffin. The ApopTag peroxidase in situ apoptosis detection kit (Millipore, Billerica, MA, USA) was used to identify apoptotic enterocytes or hepatocytes the terminal deoxynucleotidyl dUTP nick end labeling (TUNEL) analysis, as recently described [13,14].

### 2.9. Immunohistochemistry Analysis

Immunohistochemical (IHC) staining of cleaved (active) caspase-3 was conducted on paraffin-embedded liver or gut slides by using the rabbit-specific HRP/DAB (ABC) detection IHC kit (Abcam) according to the manufacturer’s instructions, as recently described [13,14].

### 2.10. Microbial 16S Sequencing and Bioinformatics

Stool samples were aseptically collected from the cecum of each mouse and rapidly frozen at −80 °C. DNA was extracted using Mag-Bind Universal Pathogen DNA Kit (Chunlab, Seoul, Korea) following the manufacturer’s protocols. DNA sequencing and bioinformatic analyses for bacterial 16S ribosomal RNA of each cecum sample were performed at the Chunlab (https://www.chunlab.com (12 March 2020).

### 2.11. RNA Extraction and Quantitative Real-Time PCR

Total RNA was extracted from the liver of each animal using Trizol (Thermo Fisher Scientific). For real-time analysis, cDNA was transcribed from a total of 600 ng of DNase I–treated RNA using the cDNA reverse-transcription kit and random primers. Quantitative real-time reverse-transcriptase polymerase chain reaction (qRT-PCR) was performed using the ABI7300 Real-Time Systems (Applied Biosystems, Forster City, CA, USA) and the SYBR Green Plus reagent system, as previously described [13,14,15]. Sequences of the forward and reverse primers specific for each target gene and GAPDH, as a control, are listed in Table S1. GAPDH was used as the reference gene.

### 2.12. Statistical Analysis and Other Methods

Data were analyzed using SPSS 26.0 program (SPSS Inc., Chicago, IL, USA), and a mean difference of *p* < 0.05 was considered significant. Different letters in the actual figures stand for significant difference between various treatment groups at *p* < 0.05 by one-way ANOVA. Once significance was recognized, Tukey’s HSD test as a post hoc analysis was conducted to compare the difference between groups. Other methods and materials not described in this study were the same as recently reported [13,14,15,16,17].

## 3. Results

### 3.1. Ellagic Acid Pretreatment Prevents the Drastic Changes in Gut Microbiota in Binge Alcohol-Exposed Mice

Our preliminary studies with three different doses of EA (e.g., *n* = 2/group for 30, 60, or 90 mg/kg/day, pretreated for 14 days) showed that daily oral administrations with EA 60 or 90 mg/kg/day significantly prevented binge alcohol-mediated gut leakiness and liver inflammation, although greater prevention was observed with higher EA dosages. Our preliminary experiment and an additional experiment (conducted at Andong National University, *n* = 4/group) consistently showed that EA or SM alone did not cause any damage to the gut and liver, as determined by H&E-stained histology and measurements of serum ALT or LPS levels (Figures S1 and S2). Another experiment (*n* = 4/group) showed that EA at 10 or 30 mg/kg/day, both of which are lower than the physiologically relevant level (approximately 60 mg/kg/day in mice), did not significantly prevent binge alcohol-mediated gut leakiness and liver damage, although EA 60 mg/kg/day showed positive effects, as shown in Figures S1 and S2. Therefore, an EA dose at 60 mg/kg/day was used in the subsequent experiments (*n* ≥ 4/group), since we are interested in studying the beneficial effects of a low, but physiologically relevant, dose of EA (in this case, 60 mg/kg/day in mice, which is close to taking one 300 mg capsule per 60 kg people), based on the calculation formula among different species [18].

**Figure 1:**
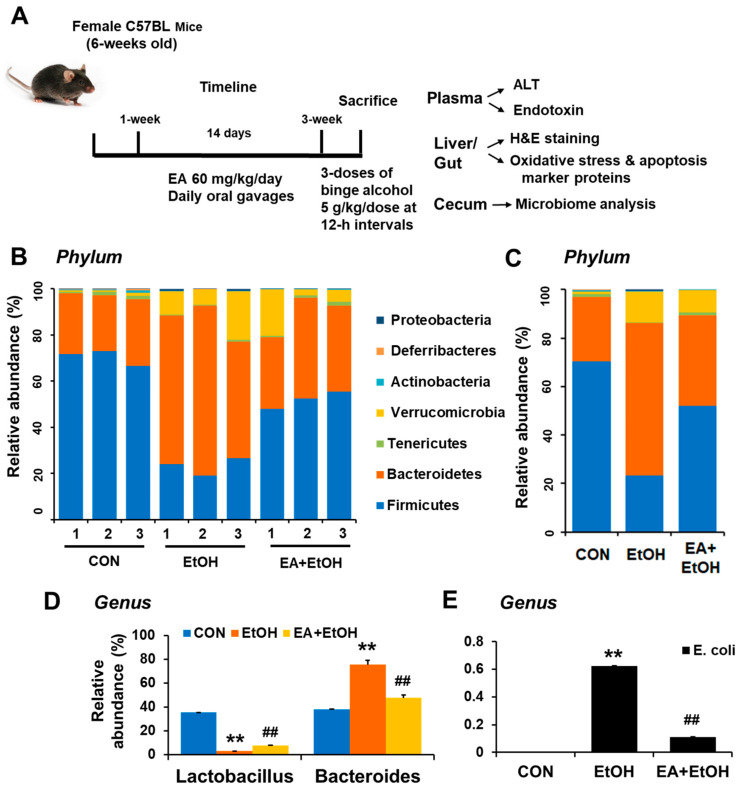
Ellagic acid pretreatment prevented binge alcohol-induced gut dysbiosis. (**A**) Summary of experiments design. (**B**,**C**) Proportional composition and abundance of various bacterial phyla to the overall gut microbiome for the three indicated groups. (**D**) The relative abundance of genus *Lactobacillus* and *Bacteroides* and (**E**) genus *E. coli* are presented for the indicated groups. ** *p* < 0.01 between EtOH and control groups; ^##^ *p* < 0.01 between EtOH vs. EA + EtOH groups. Significance of the values for each group was determined using ANOVA and Tukey’s HSD test.

Recent review articles indicate that the development and progression of alcoholic liver disease (ALD) may be closely associated with changes in the composition and abundance of gut microbiota and bacterial products [5,6,7]. Previous reports also revealed that bacteria of the phyla *Verrucomicrobia* and *Bacteroidetes* increase, whereas those of the phylum *Firmicutes* decrease in chronically alcohol-exposed mice [20,21]. To directly investigate the mechanisms behind the EA-mediated prevention of leaky gut and ALD, the composition and abundance of cecal microbiota in control versus binge alcohol-exposed mouse groups with or without EA pretreatment were compared (Figure 1A). Gut microbiome sequencing analyses showed no difference in phylum composition between control and EA pretreatment groups despite the significant alterations in their quantities (Figure 1B). In binge alcohol-exposure mice, the amounts of *Verrucomicrobia* and *Bacteroidetes* increased, whereas *Firmicutes* abundance decreased at the phylum level (Figure 1C). As one of the most abundant genes, *Bacteroides* showed the greatest elevation in binge alcohol-exposed mice (Figure 1C,D and Supplementary Figure S1). However, *Bacteroides* population significantly decreased in EA-pretreated mice (Figure 1C,D). Interestingly, *Lactobacillus* abundance markedly decreased in the alcohol-exposed group but was enriched in EA pretreated mice, although its population was much less than that of the control (Figure 1D and Figure S3). In contrast, *E. coli* abundance was markedly elevated in binge alcohol-exposed mice, while its amounts were significantly reduced in EA pretreated mice (Figure 1E). These results demonstrate that EA pretreatment was likely to block or retard the gut dysbiosis caused by binge alcohol-exposure, thus leading to the significant prevention of leaky gut and endotoxemia observed in binge alcohol-exposed rodents [13,14].

### 3.2. Ellagic Acid Pretreatment Averts the Increased Levels of Plasma Endotoxin and Intestinal TNF-α and IL-1β Proteins in Binge Alcohol-Exposed Mice

**Figure 2:**
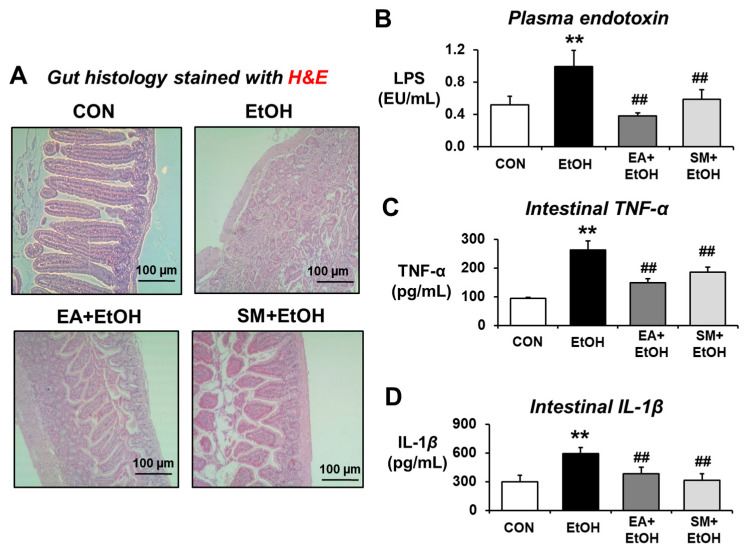
Ellagic acid pretreatment averted binge alcohol-mediated gut damage, endotoxemia, and elevated levels of intestinal TNF-α and IL-1β. (**A**) Representative H&E of formalin-fixed ileum sections for control (CON), ethanol (EtOH), EA (ellagic acid) + EtOH, or SM (silymarin) + EtOH mouse group. (**B**–**D**) Representative levels of (**B**) plasma endotoxin, (**C**) TNF-α, and (**D**) IL-1β in the ileum lysates from the indicated groups are presented. Data represent means  ± SD. ** *p* < 0.01 between EtOH and control groups; ^##^ *p* < 0.01 between EtOH vs. EA + EtOH or SM + EtOH groups. Significance of the values for each group was determined using ANOVA and Tukey’s HSD test.

Our recent reports showed that increased leaky gut accompanied by gut microbiome changes can contribute to systemic endotoxemia with elevated levels of endotoxin LPS and inflammatory liver disease in rodents exposed to binge alcohol [14] or fructose in drinking water [15]. In addition, a recent report showed that altered gut microbiota with different bacterial antigens and metabolites can stimulate gut leakiness and peripheral immune activation in alcohol-fed mice [22]. Thus, the preventive effects of EA on binge alcohol-induced endotoxemia and inflammation marker proteins were determined. H&E-stained histology revealed the disorganization and detachment of many intestinal epithelial cells with abnormal villi shapes in alcohol-exposed mice compared to those of controls containing normal patterns of gut villi structure and organization (Figure 2A). Both EA and silymarin (SM) pretreatment significantly prevented the abnormal villi structure caused by ethanol exposure, although EA-exposed gut looks slightly better than that of silymarin (SM)-pretreated mice (Figure 2A). Consistently, binge alcohol exposure markedly elevated the plasma endotoxin concentration compared to the control mice, whereas EA or SM pretreatment significantly attenuated the alcohol-related elevation of endotoxin (Figure 2B). Similarly, elevated levels of intestinal inflammation marker proteins TNF-α and IL-1β were observed in alcohol-exposed mice compared to those of the control, while pretreatment with EA or SM significantly prevented the elevation of these pro-inflammatory cytokines (Figure 2C,D, respectively).

### 3.3. Ellagic Acid Pretreatment Reduced the Gut Oxidative Stress Markers in Binge Alcohol-Exposed Mice

**Figure 3:**
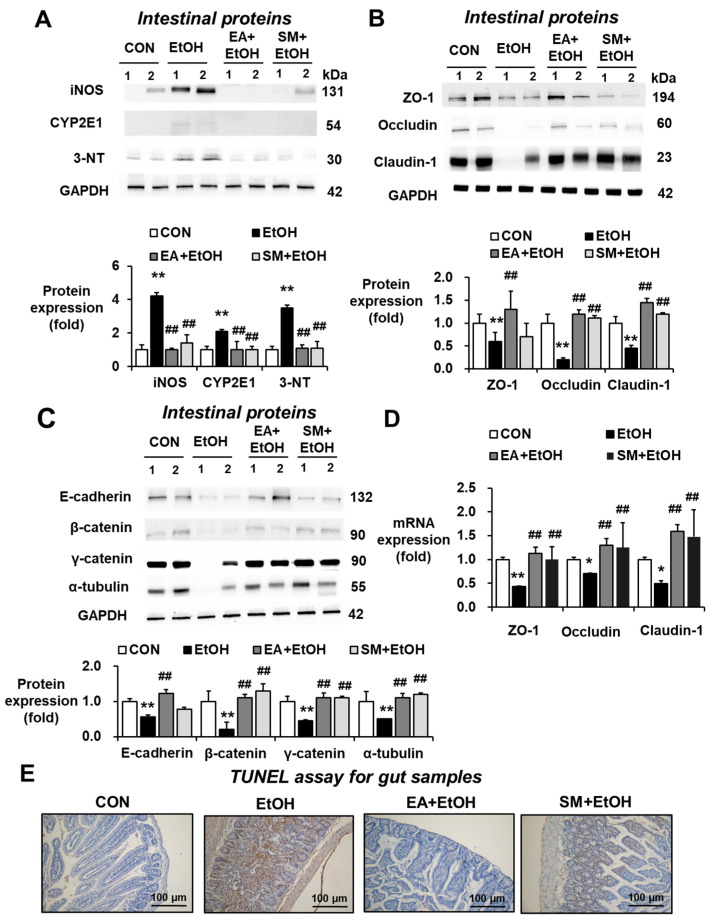
Ellagic acid pretreatment prevented changes in oxidative stress markers, gut TJ/AJ proteins, and apoptosis rates in binge alcohol-exposed mice. (**A**) Representative levels of intestinal iNOS, CYP2E1, and 3-NT in the indicated groups are presented. The levels of (**B**) intestinal TJ proteins, (**C**) AJ proteins, and (**D**) their mRNA transcripts in the indicated groups are presented. Densitometric quantitation of each immunoreactive protein, relative to a reference control GAPDH (**A**–**C**), is shown. Data represent means  ± SD. (**E**) Representative images of TUNEL assay for the indicated groups are presented. * *p* < 0.05, ** *p* < 0.01 between EtOH and control groups; ^##^ *p* < 0.01 between EtOH vs. EA + EtOH or SM + EtOH groups. Significance of the values for each group was determined using ANOVA and Tukey’s HSD test.

Numerous studies have demonstrated that oxidative stress indicated by the elevated levels of CYP2E1, iNOS, and nitrated proteins plays a key role in promoting alcohol-mediated intestinal barrier dysfunction and fatty liver disease through the gut–liver axis. Our current results showed that binge alcohol significantly elevated the amounts of intestinal iNOS, CYP2E1, and 3-NT, whereas EA or SM pretreatment significantly reduced the elevated levels of these oxidative stress marker proteins in alcohol-exposed mice (Figure 3A). Therefore, it is likely that EA or SM pretreatment prevented alcohol-mediated gut leakiness by inhibiting the elevated oxidative stress.

### 3.4. Ellagic Acid Pretreatment Prevented Altered Levels of Gut Tight Junction, Adherent Junction, and Apoptosis Marker Proteins in Binge Alcohol-Exposed Mice

The markedly decreased expression of intestinal tight junction (TJ) and adherent junction (AJ) proteins associated with the gut barrier function was observed in mouse and rat models of binge alcohol-induced leaky gut, endotoxemia, and fatty liver [13,14,15]. Therefore, the effects of EA or SM pretreatment on the levels of intestinal TJ and AJ proteins were quantitatively measured by immunoblot analyses. Binge alcohol exposure significantly reduced the levels of AJ proteins (e.g., ZO-1, claudin-1, and occludin in Figure 3B) and AJ proteins (e.g., E-cadherin, β-catenin, γ-catenin, and α-tubulin in Figure 3C), whereas EA or SM pretreatment significantly prevented the decrements of these TJ and AJ proteins, although the preventive effects of EA on gut TJ/AJ proteins appeared better than those of SM (Figure 3B,C). In addition, EA or SM pretreatment restored the decreased mRNA levels of intestinal ZO-1, claudin-1, and occludin in ethanol-exposed mice (Figure 3D). Consistently, TUNEL analyses revealed that alcohol exposure elevated the rates of enterocyte apoptosis, while as EA or SM treatment reduced the apoptosis rates increased by binge alcohol exposure (Figure 3E). Taken together, these results showed that binge alcohol exposure decreased the levels of gut TJ and AJ proteins and the increased apoptosis of enterocytes, contributing to elevated leaky gut and endotoxemia. However, pretreatment with EA or SM significantly averted the decreased amounts of the intestinal TJ and AJ proteins and prevented the death of enterocytes following binge alcohol exposure.

### 3.5. Ellagic Acid Pretreatment Prevented Alcohol-Mediated Hepatic Fat Accumulation, Plasma ALT, and Hepatic Triglyceride Levels

**Figure 4:**
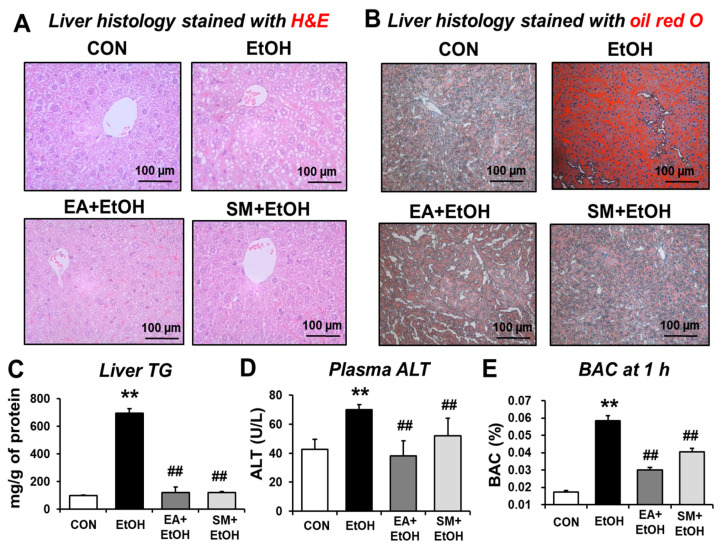
Ellagic acid pretreatment attenuated binge alcohol-induced fatty liver and acute liver injury in mice. (**A**,**B**) Representative H&E (**A**) or Oil Red O (**B**) staining of formalin-fixed or frozen liver sections for control (CON), ethanol (EtOH), EA + EtOH, or SM + EtOH mouse groups. (**C**–**E**) The levels of (**C**) hepatic triglyceride (TG), (**D**) plasma ALT, and (**E**) BAC are shown. Data represent means ± SD. ** *p* < 0.01 between EtOH and control groups; ^##^ *p* < 0.01 between EtOH vs. EA + EtOH or SM + EtOH groups. Significance of the values for each group was determined using ANOVA and Tukey’s HSD test.

Binge alcohol intake can stimulate gut leakiness, contributing to the increased levels of LPS, which activates liver Kupffer cells and hepatic steatosis, a major manifestation of ALD in humans and animal models. To evaluate the preventive effect of EA on fat accumulation and/or inflammatory liver injury, histological analyses were conducted. H&E and oil red O staining showed markedly elevated fat accumulation in the liver after binge alcohol-exposure (Figure 4A,B, respectively). However, EA or SM treatment prevented hepatic fat accumulation, and inflammatory foci increased after binge alcohol-exposure (Figure 4A,B). The levels of hepatic triglyceride (TG) and plasma ALT were also increased by binge alcohol-exposure (Figure 4C,D). In addition, EA or SM treatment significantly attenuated the elevated levels of haptic TG and plasma ALT in alcohol-exposed mice. Furthermore, EA or SM treatment significantly reduced elevated blood alcohol concentration (BAC) 1 h after the last oral ethanol administration, although the effect of EA was significantly better than that of SM (Figure 4E). However, the body weight gain and the liver/body weight ratio were unchanged and similar in all groups (Supplementary Table S2). These results suggest that EA or SM significantly prevented the increments of hepatic fat accumulation, plasma ALT, and hepatic TG levels in a mouse model for alcohol-induced leaky gut and acute liver injury.

### 3.6. Ellagic Acid Pretreatment Attenuated the Hepatic Oxidative Stress Marker Proteins in Binge Alcohol-Exposed Mice

**Figure 5:**
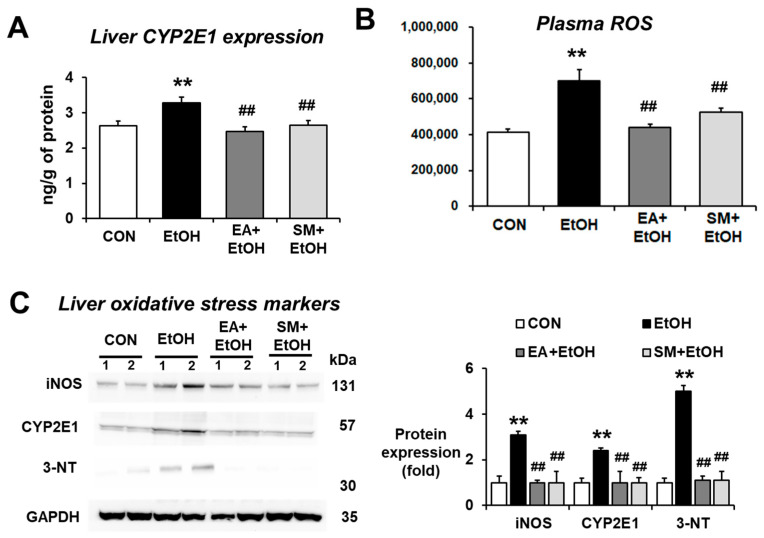
Ellagic acid pretreatment prevented the elevation of oxidative stress and liver pro-oxidant marker proteins in binge alcohol-exposed mice. (**A**,**B**) The levels of hepatic CYP2E1 expression and plasma ROS in the indicated groups are presented. (**C**) The levels of hepatic CYP2E1, iNOS, and nitrated proteins detected by anti-3-NT antibodies in the indicated groups are presented. Densitometric quantitation of the immunoblots for each protein relative to GAPDH is shown. Data represent means ± SD. ** *p* < 0.01 between EtOH and control groups; ^##^ *p* < 0.01 between EtOH vs. EA + EtOH or SM + EtOH groups. Significance of the values for each group was determined using ANOVA and Tukey’s HSD test.

Ethanol-inducible CYP2E1 plays an important role in producing alcohol-related oxidative stress in many tissues, including the liver and intestines, contributing to alcohol-induced fatty liver and gut leakiness [23,24,25,26]. In order to investigate the effects of EA on CYP2E1-related oxidative stress parameters, the levels of hepatic CYP2E1 protein expression were determined. As expected, significantly increased levels of hepatic CYP2E1 protein were observed in alcohol-exposed mice (Figure 5A,C). In contrast, EA or SM treatment attenuated the elevated levels of CYP2E1 protein after binge alcohol exposure. Other oxidative stress marker proteins, such as inducible nitric oxide synthase (iNOS) and nitrated proteins as probed with anti-3-NT antibodies, were significantly increased in alcohol-exposed mice (Figure 5C), whereas their elevations were significantly blunted in EA- or SM-treated mice (Figure 5C). 

CYP2E1-mediated metabolism can produce ROS, which can promote ALD [23,24,25,26] and non-alcoholic fatty liver disease (NAFLD) [27,28]. Our results showed that the levels of plasma ROS were significantly elevated in alcohol-exposed mice (Figure 5B), while EA or SM pretreatment significantly attenuated the increased levels of plasma ROS following binge alcohol exposure. These results demonstrate that EA or SM significantly prevented the elevated levels of systemic oxidative stress and liver pro-oxidant marker proteins in alcohol-exposed mice. 

### 3.7. Ellagic Acid Pretreatment Prevented the Elevated Hepatic Apoptosis Marker Proteins in Binge Alcohol-Exposed Mice

**Figure 6:**
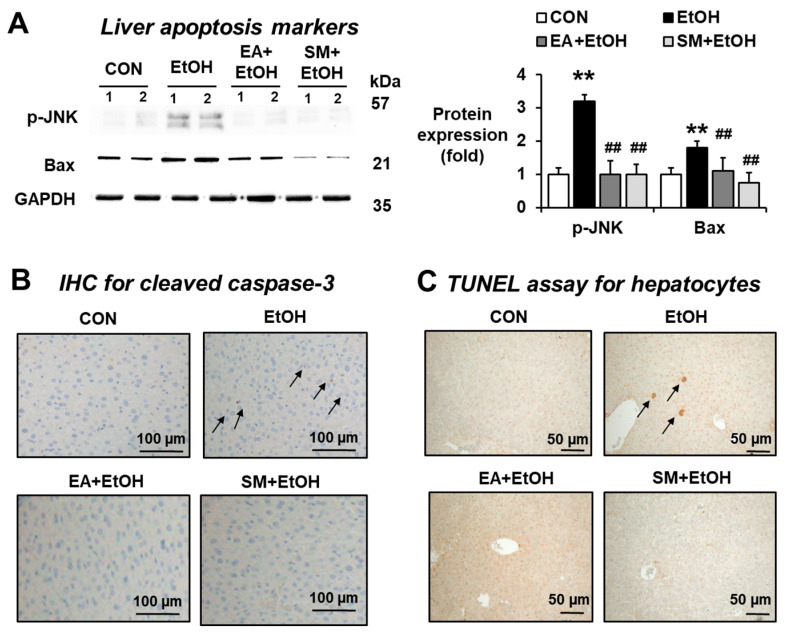
Ellagic acid pretreatment attenuated the elevation of hepatic apoptosis marker proteins in binge alcohol-exposed mice. Representative levels of apoptosis marker proteins (**A**) p-JNK, Bax, (**B**) IHC staining for cleaved caspase-3 and (**C**) TUNEL assay in the indicated groups are shown. The arrows indicate positive hepatic cells stained with (**B**) cleaved caspase-3 and (**C**) TUNEL analysis. Densitometric quantitation of the immunoblots for each protein relative to GAPDH is shown. ** *p* < 0.01 between EtOH and control groups; ^##^ *p* < 0.01 between EtOH vs. EA + EtOH or SM + EtOH groups. Significance of the values for each group was determined using ANOVA and Tukey’s HSD test.

To investigate whether EA prevents the elevated hepatocyte apoptosis in the binge alcohol-exposed mice, we performed Western blot analysis for p-JNK and Bax as apoptosis marker proteins, immunohistochemical (IHC) staining for cleaved (active) caspase-3, and TUNEL assay. Our results showed that apoptosis marker proteins p-JNK and Bax were slightly but significantly increased in the alcohol-exposed mice compared to controls, but their elevations were significantly prevented in the EA- or SM-treated mice (Figure 6A). In addition, both cleaved caspase-3 IHC staining and TUNEL assay revealed that the levels of hepatocyte apoptosis markers were significantly elevated in the alcohol-exposed mice (Figure 6B,C, respectively). In addition, EA or SM treatment blunted the elevated rates of hepatic apoptosis in binge alcohol-exposed mice (Figure 6B,C). Taken together, these results show that EA or SM treatment prevents hepatocyte apoptosis elevated in binge alcohol-exposed mice.

## 4. Discussion

It is well-established that alcoholic liver disease (ALD), caused by excessive drinking, is a major disease that can be prevented by simply abstaining from alcohol consumption. Recent epidemiological reports (CDC-2011) revealed that binge alcohol drinking is responsible for ~77% of the socioeconomic costs associated with alcohol misuse [1,29]. ALD is one of the major alcohol-related pathological manifestations. It was reported that a significant portion (30~40%) of people with alcohol use disorder would die within 1 month after the first clinical diagnosis of alcoholic hepatitis [11]. Unfortunately, there is no FDA-approved drug for treating patients with alcoholic hepatitis or fibrosis/cirrhosis at this moment, although many agents are being evaluated in randomized clinical tests [30,31]. Therefore, there is an urgent need for developing a safe and effective drug in treating patients with alcoholic hepatitis or fibrosis.

It is well-established that CYP2E1-related oxidative ethanol metabolism [13,23,24,25,26] and mitochondrial dysfunction with inactivated and/or decreased levels of the electron transport chain proteins can elevate ROS production, contributing to increased oxidative stress and tissue injury in alcohol-exposed rodents and people with AUD [14,32,33,34,35,36]. Increased oxidative and nitrosative/nitrative stress, through elevated and/or activated liver CYP2E1, NADPH oxidase, and iNOS, can play an important role in promoting ALD and the activation of hepatic stellate cells for liver fibrosis, since genetic deletion or using specific inhibitors of these pro-oxidant enzymes significantly attenuated the levels of ALD in various rodent and cell culture models [24,35,36,37,38,39,40]. In addition, transgenic mice with over-expressed CYP2E1 or humanized CYP2E1 knock-in mice increased the severity of ALD [25,39]. Furthermore, ALD could be indirectly promoted through elevated leaky gut and endotoxin LPS, which interacts with and stimulates TLR4-mediated inflammation signaling pathway in the liver. In fact, our laboratory recently reported that binge alcohol caused leaky gut, endotoxemia, and acute inflammatory liver injury via increased the apoptosis of gut enterocytes and post-translational modifications of intestinal TJ and AJ proteins, leading to their degradation [14]. The markedly decreased levels of many TJ and AJ proteins in alcohol-exposed rats were confirmed by mass-spectral analysis of the purified intestinal junctional complex proteins. Binge alcohol-mediated gut barrier dysfunction and acute liver injury were observed in rats and wild-type mice but not in the corresponding *Cyp2e1*-null mice on Svj129 background. Furthermore, treatment with an antioxidant *N*-acetylcysteine or a selective inhibitor of CYP2E1 chlormethiazole (CMZ) suppressed the levels of intestinal CYP2E1 and alcohol-mediated endotoxemia [14,23], suggesting the important role of intestinal CYP2E1 and oxidative stress in promoting gut leakiness and inflammatory liver injury.

**Figure 7:**
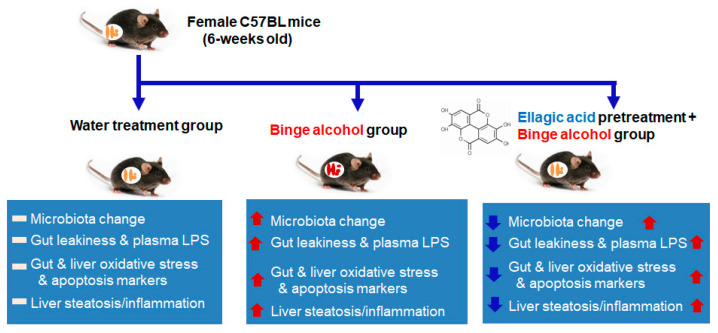
Summary of the preventive effects of ellagic acid against alcohol-Induced oxidative stress, gut leakiness, endotoxemia, and inflammatory fatty liver through gut microbiome changes and the suppression of CYP2E1. The up (red) and down (blue) arrows represent an increment by binge alcohol exposure and a decrement of each indicated parameter, respectively, by EA pretreatment.

Many synthetic and natural compounds can suppress or inhibit the CYP2E1 activities. For instance, CMZ was shown to inhibit CYP2E1 at the transcriptional level [14], while insulin was shown to suppress CYP2E1 by transcriptional and posttranslational mechanisms [41]. In addition, many natural products, such as indole-3-carbinol [34], diallyl sulfide [42], phenethyl isothiocyanate [43], resveratrol [44,45], berberine [46], melatonin [47], lycopene [48], ellagic acid [49], silibinin [50], silymarin [51], gallic acid [51], walnut phytochemicals [52], etc., can suppress CYP2E1 activity and thus, fatty liver disease. It is thus possible that many other antioxidant phytochemicals, not listed here or yet reported, can suppress CYP2E1 and ALD [6]. We also showed that pomegranate extract, albeit being used at a pharmacologically high dose, prevented binge alcohol-mediated leaky gut and liver injury by suppressing the intestinal and hepatic CYP2E1 levels [13]. The protective effects of pomegranate extract were also observed in both T84 human colon cancer cells and AML12 mouse hepatocytes after these cells were incubated with EA, which significantly attenuated the changes in the levels of gut CYP2E1, TJ/AJ proteins, and epithelial barrier dysfunction following ethanol exposure [13]. One of the main components of pomegranate is EA. However, it is still unknown about the beneficial effect of physiologically relevant doses of pomegranate extract or its major component, EA. Therefore, in this study, we aimed to study the beneficial effect of a physiologically relevant dose (approximately 60 mg/kg/day in mice) of EA on binge alcohol-mediated leaky gut and inflammatory liver injury. Our results showed that EA could prevent binge alcohol-mediated acute liver injury through various mechanisms. For instance, as a known antioxidant, EA could inhibit alcohol-induced oxidative cell damage by increasing the antioxidant levels, scavenging free radicals, and stabilizing cell membranes [12,53,54]. In addition, EA was reported to protect hepatocytes by regulating the activity of CYP450 enzymes, including CYP2E1, by decreasing the transformation of many xenobiotics, including a procarcinogen nitrosodimethylamine (NDMA) to toxic metabolites, and preventing oxidative injury and NDMA-mediated mutagenesis [13,54,55]. Other reports also demonstrated that EA has anti-inflammatory properties by reducing the expression of pro-inflammatory and pro-fibrogenic cytokines like interleukins (IL-1α, IL-6, IL-8), TNF-α, and TGF-β, which are involved in alcohol-induced inflammation and fibrosis [53,54,55]. Despite these and other reports [12,53,54,55,56,57,58,59], to our knowledge, the beneficial effects of EA on preventing binge alcohol-mediated gut dysbiosis, decreased gut TJ/AJ proteins, and elevated apoptosis of intestinal enterocytes, leading to gut leakiness, endotoxemia, and acute liver injury through the gut–liver axis, has not been systematically studied. For instance, despite the previous reports about the beneficial effects of EA against alcohol-associated lipid peroxidation and hepatotoxicity [53,54,55,56,57], the direct effects of physiological doses of EA or pomegranate extract on the alcohol-induced changes in the composition and abundance of gut microbiota were unknown. Therefore, it is important to assess the beneficial effects of a physiologically-relevant dose of EA on alcohol-induced gut microbiota changes, leaky gut, and liver injury in a rodent model and study the underlying mechanisms of protection at the molecular levels, as outlined (Figure 7).

Chronic and/or binge alcohol abuse is known to cause intestinal dysbiosis and bacterial overgrowth, leading to elevated intestinal barrier dysfunction, peripheral inflammation, and liver toxicity [5,6,7,8,22]. In fact, the severity of alcohol-induced lesions is positively correlated with increased levels of endotoxin (LPS) in the blood of rodents and human patients [10,60]. In alcohol-fed mice, bacteria of the phyla *Verrucomicrobia* and *Bacteroidetes* increase, whereas those of the phylum *Firmicutes* and *Akkermansia* decrease [21]. In alcoholic patients, *Proteobacteria* and *Firmicute* phyla increase; however, this increase seems to depend on the stage of liver disease [61]. A recent study reported that the numbers of cytolysin-positive *Enterococcus faecalis* correlate with the severity of alcoholic hepatitis and mortality [62]. Interestingly, the intestinal overgrowth of *Klebsiella pneumoniae* causes fatty liver disease because these bacteria produce alcohol endogenously, even in the absence of alcohol consumption [63]. Our current data showed that bacteria of the phyla *Verrucomicrobia* and *Bacteroidetes* increase, whereas those of the phylum *Firmicutes*, including *Lactobacillus*, decrease in binge alcohol-exposed mice, and the abundance changes in these bacterial phyla were prevented by EA pretreatment for 2 weeks (Figure 1). Our current results of decreased *Lactobacillus* in alcohol-exposed mice may explain the reasons for the beneficial effects of the administration of *Lactobacillus* against alcohol-mediated endotoxemia and fatty liver disease [64,65]. Our results show that EA pretreatment can also exhibit another protective mechanism against leaky gut and inflammatory liver injury by significantly preventing the alcohol-mediated gut dysbiosis, resulting in the maintenance of healthy gut microflora similar to those in control mice (Figure 7).

Elevated intestinal and hepatic CYP2E1 are important for promoting alcohol-induced gut leakiness and hepatic steatosis, respectively [13,23,24,25,26], possibly via oxidative/nitrative stress and the apoptosis of parenchymal cells. The present data shows that intestinal CYP2E1 and oxidative stress seem to play an essential role in the gut leakiness, as evidenced by significantly decreased levels of gut TJ/AJ proteins, elevated levels of serum endotoxin, and prominent histological changes in the small intestine of binge alcohol-exposed mice. However, the prevention from the alcohol-induced gut dysbiosis, which can cause leaky gut and local inflammation through the microbial products and abnormal metabolites [22], by EA pretreatment could also contribute to the protection from alcohol-induced intestinal barrier dysfunction and liver injury, despite the low bioavailability of EA [6]. In addition, EA or its gut metabolite urolithic acid (UA) can work as antioxidants [12,53,54,58,59]. This fact suggests that EA and/or its gut metabolite UA could reduce the oxidative stress by directly neutralizing various ROS and reactive nitrogen species. Finally, EA treatment attenuated apoptosis marker proteins in binge alcohol-exposed mice possibly neutralizing the *p*-JNK mediated cell death signaling pathway, which is stimulated under increased oxidative stress [33,66]. Our preliminary results showed that pretreatment with a physiologically relevant dose of EA (60 mg/kg/day) also prevented a mouse model of colitis caused by dextran sulfate sodium (DSS) treatment for 7 days by following the established protocols [67]. In this case, EA pretreatment for 7 days significantly prevented DSS-mediated gut damage, endotoxemia, and acute liver injury by attenuation of the elevated plasma ALT, AST, and endotoxin (LPS) and the restoration of abnormal gut and liver histology in DSS-exposed mice (preliminary data only shown to the reviewers for reviewing purposes). These results suggest that EA at a physiologically relevant dose can be possibly used as a safe agent against many inflammatory diseases.

## 5. Conclusions

In this study, we investigated the hypothesis whether EA, a naturally occurring antioxidant and a major component of pomegranate, can prevent the binge alcohol-mediated gut dysbiosis at multiple taxonomic levels, leading to decreased leaky gut and acute liver injury. Our results showed that changes in gut microflora can influence gut leakiness, liver steatosis, and inflammation following binge alcohol exposure through the gut–liver axis, as described [6]. In addition, this study showed that intestinal and hepatic CYP2E1, induced by binge alcohol exposure, plays an important role in promoting oxidative stress, gut leakiness, and endotoxemia, all of which could be blunted by a physiologically relevant dose of antioxidant EA. Our results showed that the beneficial effects of EA against alcohol-mediated oxidative stress, leaky gut, endotoxemia, and liver injury were comparable to those of silymarin, which is widely-used as a dietary supplement for treating liver disease [68]. Although EA dosage (60 mg/kg/day) was much lower than that of silymarin (SM, 200 mg/kg/day), used a positive control, our data showed that EA may be better and more potent than SM on concentration basis in regulating the levels of gut iNOS & CYP2E1 (Figure 3A), TJ/AJ proteins (Figure 3B,C), serum ALT (Figure 4D), BAC (Figure 4E), and ROS (Figure 5B). Although we do not know the reasons for the different effects of EA versus SM on binge alcohol-mediated leaky gut and acute liver injury, the final outcomes could be affected by the different rates of their absorption, metabolism, gut microbial changes, antioxidant capacity, etc. Nonetheless, EA may be used as a safe dietary supplement to effectively manage alcohol-mediated gut dysbiosis, leakiness, endotoxemia, and fatty or inflammatory liver disease.

## Data Availability

The data presented in this study are available in the article.

## Supplementary Materials

The following are available online at https://www.mdpi.com/article/10.3390/antiox10091386/s1, (Figures S1 and S2): Ellagic acid pretreatment at 60 mg/kg/day, but not with 10 or 30 mg/kg/day, prevented binge alcohol-induced liver injury in mice (*n* = 4/group) (Figure S1) or gut damage and endotoxemia (Figure S2); Figure S3: Changes in top 10 most abundant genera in alcohol-exposed mice with or without EA pretreatment; Table S1: Sequences of the specific oligonucleotide primers for each target gene used in the real-time PCR; Table S2: Body weight gain and ratio of liver/body weight in mice with different treatments.
