# Supplementary material for: Ellagic Acid Prevents Binge Alcohol-Induced Leaky Gut and Liver Injury through Inhibiting Gut Dysbiosis and Oxidative Stress
Source: Antioxidants (Basel). 2021 Aug 30;10(9):1386. doi: 10.3390/antiox10091386 (PMC8465052; doi:10.3390/antiox10091386)
Supplement: Supplementary file 1 [file antioxidants-10-01386-s001.zip › antioxidants-1327369-supplementary.pdf]

Table S1. Sequences of the specific primers used in qRT-PCR analysis.

Gene	Sequence (5' → 3')
ZO-1	F: 5'-AGCATCCACGTGCTGTCTC-3'<br>R: 5'-GATCATCTTGCCAGTGAATGAG-3'
Claudin1	F: 5'-TGCCAACCTTGTGGTATCAGCCA-3'<br>R: 5'-TGAAGACACAGAGAAGCAATCC-3'
Occludin	F: 5'-ATGAGAAGCGGGAGTCTG AA-3'<br>R: 5'-ACGGTGCATTAATCAATTTC-3'
GAPDH	F: 5'-TCCACTCACGGCAAATTCAACG -3'<br>R: 5'-TAGACTCCACGACATACTCAGC-3'

Reference for GAPDH as a stable gene: Wang S, Wang J, Lv X. Selection of reference genes for expression analysis in mouse models of acute alcoholic liver injury. *Int J Mol Med* 41: 3527-3536, 2018; PMID:29512759.

Table S2. Body weight gain and ratio of liver/body weight in mice with different treatments

Groups	CON	EtOH	EA+EtOH	SM+EtOH
**Final body weight (g)**	18.23±1.12	17.753±0.86	17.889±0.78	17.92±0.84
**Liver weight, g**	0.98±0.05	0.94±0.06	0.89±0.063	0.93±0.06
**LW/BW, %**	5.39±0.39	5.32±0.40	4.95±0.40	5.20±0.48

Data represents means ± SD.

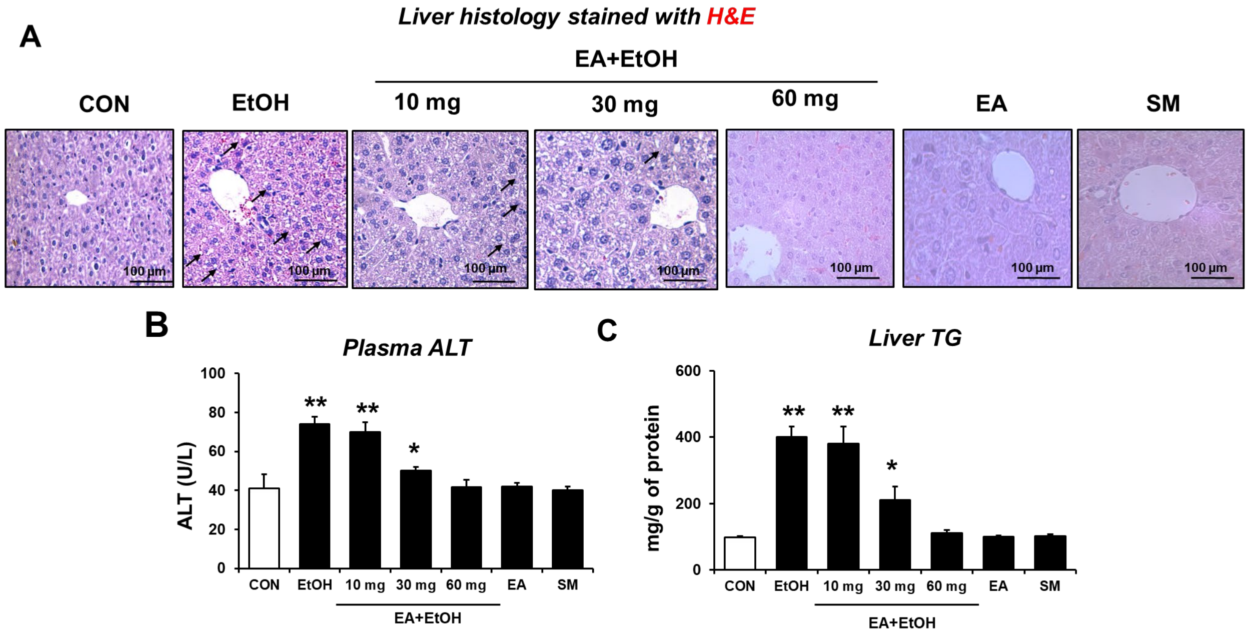

Figure S1. Ellagic acid pretreatment at the lower doses did not prevent binge alcohol-induced liver injury in mice. (A, B) Representative liver histology stained with H/E (A) for control (CON) or ethanol (EtOH) in the absence or presence of an indicated dose of EA pretreatment (10, 30, or 60 mg/kg/day for 14 consecutive days), EA alone, or SM alone in mice. (B-C) The levels of (B) plasma ALT and (C) hepatic triglyceride (TG) are shown. Data represent means ± SD. *$P < 0.05$, **$P < 0.01$.

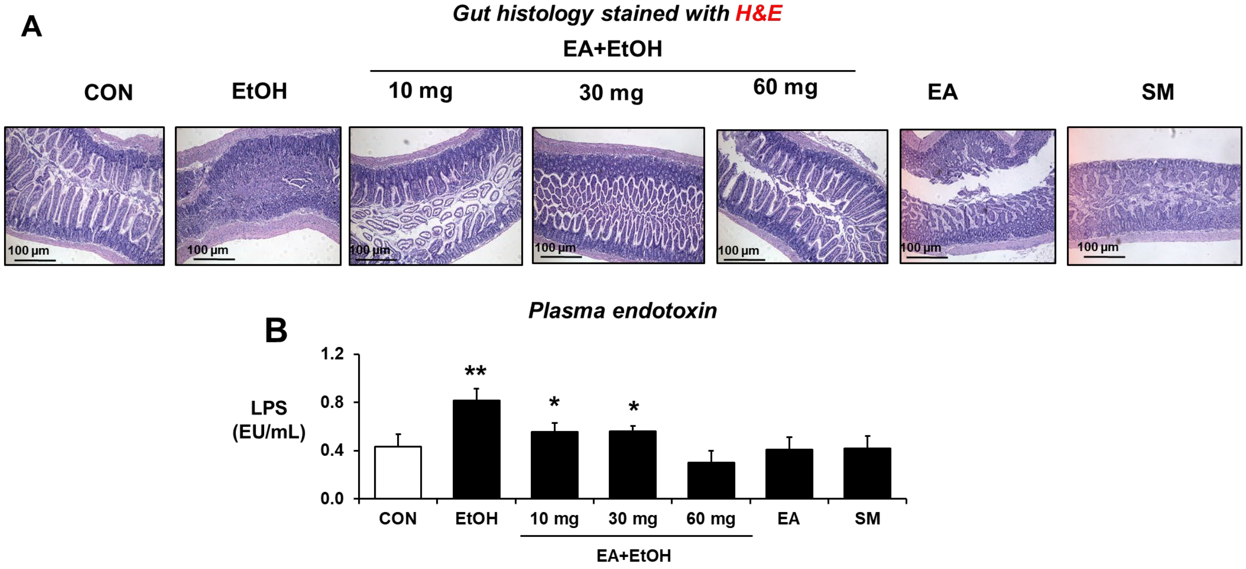

Figure S2. Ellagic acid pretreatment at lower doses did not attenuate binge alcohol-mediated gut damage and endotoxemia. (A) Representative histology of ileum stained with H/E for control (CON) or ethanol (EtOH) in the absence or presence of an indicated dose of EA pretreatment (10, 30, or 60 mg/kg/day for 14 consecutive days), EA alone, or SM alone in mice. (B) Representative levels of plasma endotoxin in the indicated groups are presented. Data represent means ± SD. ***P* < 0.01, **P* < 0.05.

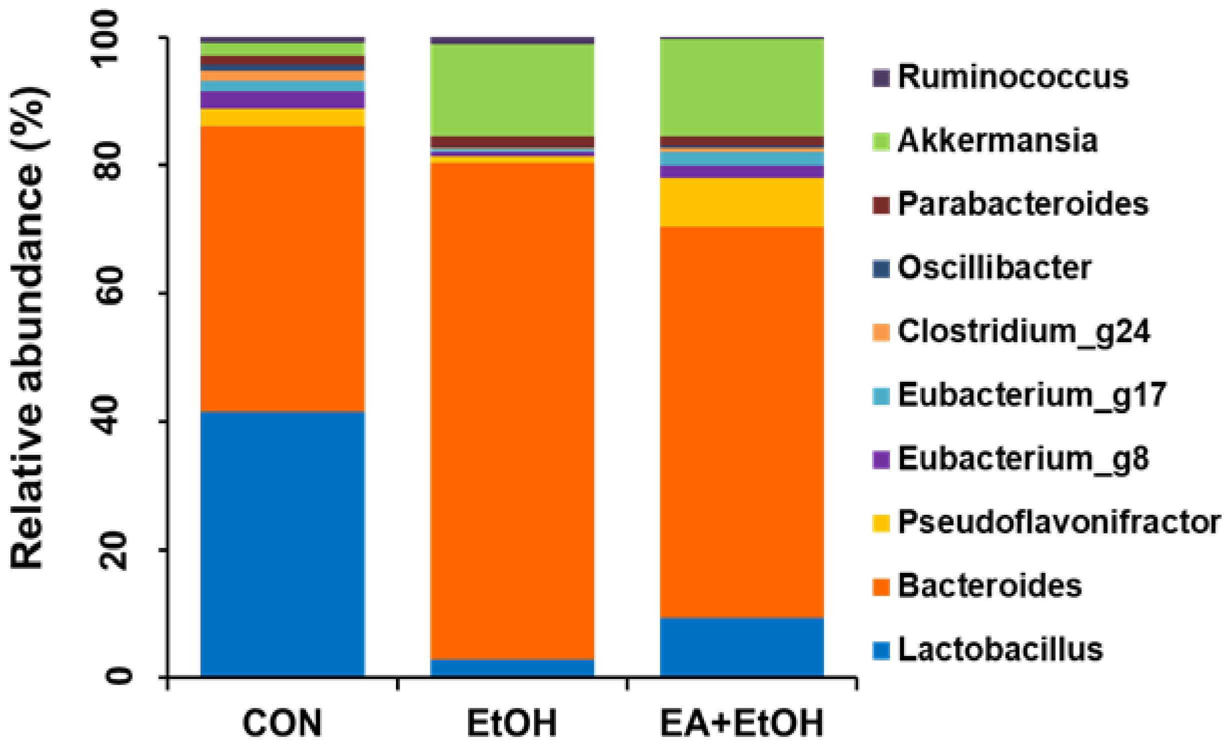

Figure S3. Ellagic acid pretreatment prevents binge alcohol-mediated changes in the top 10 most abundant bacterial genera.
